# Surveys on migration aspirations, plans and intentions: a comprehensive overview

**DOI:** 10.12688/openreseurope.15800.1

**Published:** 2023-09-11

**Authors:** Mathilde Bålsrud Mjelva, Jørgen Carling

**Affiliations:** 1 Peace Research Institute Oslo, Oslo, Norway

**Keywords:** migration, survey, migration aspirations, migration intentions, migration plans, survey methodology, data compilation, systematic review

## Abstract

Survey data on migration aspirations, plans and intentions is important for understanding the drivers and dynamics of migration. Such data has been collected since the 1960s but has expanded massively in recent decades. This paper provides the first comprehensive overview of existing survey data in an inventory of 212 surveys with recorded metadata on geographic and temporal coverage, survey population, sample size, and other characteristics. ‘A survey’ is not always a clear-cut unit of analysis, but we adopted procedures that enable systematic comparisons, and identified surveys through systematic searches and follow-up investigation. The paper has three objectives. First, it facilitates reuse of survey data and secondary analysis, albeit with limitations in data access, which we document. Second, it helps consolidate a sprawling field and thereby contribute to methodological and theoretical strengthening. Third, it informs debates on the ethics, politics and biases of data collection by documenting broad patterns in the body of knowledge. The inventory of survey data on migration aspirations and related concepts gives migration researchers a new tool for locating existing data and strengthening the foundations for collecting new data.

## Plain language summary

Why do some people wish to leave their current homes and others not? What are the origins of migration plans, hopes and dreams? Researchers have studied these questions through survey data for decades. This article presents an overview of surveys on migration aspirations, plans, and intentions collected since the 1960s. Our inventory of surveys contains information on the surveys’ geographic focus, data collection period, survey population and methodology. By providing such an overview of surveys on migration aspirations, plans, and intensions, this paper facilitates reuse of the survey data and exposes patterns and biases in the focus of this research over the past decades. As such, the inventory of surveys helps researchers locate existing survey data and plan new data collection.

## 1 Introduction

The study of migration has benefitted from collecting and analysing survey data on individuals’ thoughts and feelings regarding the possibility of moving elsewhere. In line with recent developments in migration theory, we use ‘migration aspirations’ as the umbrella term to cover these mental constructs in their various forms, including desires, intentions, plans and expectations for migration ( Carling, 2002; Carling & Schewel, 2018; de Haas, 2021; Koikkalainen & Kyle, 2016).

Within migration studies, survey data on migration aspirations complements population data by allowing for incorporation of fine-grained information on attitudes and behaviour. Moreover, it complements survey data on migrants, which offers limited insight on the drivers of migration because it is sampled on the dependent variable.

Data on migration aspirations has in part been used in attempts to predict or forecast migration flows ( Tjaden *et al.*, 2019). Even though most prospective migrants face daunting obstacles and end up staying, variations in the incidence of migration can shed light on the evolution of migration flows. Moreover, there are additional reasons for studying migration aspirations ( Aslany *et al*., 2021; Carling, 2019). First, if we want to understand what *motivates* migration, it is insufficient to study actual migration. Factors such as poverty, corruption, crime, or environmental degradation could affect peoples’ wish to move elsewhere ( Aslany *et al*., 2021; Czaika & Reinprecht, 2022). Whether or not they lead to people crossing borders is a separate issue, governed not least by restrictive migration policies and other obstacles. Visa restrictions have, for instance, been found to decrease emigration ( Czaika & de Haas, 2017). Second, migration aspirations could affect behaviour in other ways than migration, especially when the desire to leave remains unfulfilled for many years. People who wait for a chance to leave could, for instance, be less inclined to invest in local livelihoods, skills or relationships, with consequences for their own lives and societies.

From a policy perspective, insights on migration aspirations are essential for influencing migration flows and reducing the negative consequences of migration. As we will show, many surveys specifically target health workers and medical students and could provide insights that help stem the loss of human capital through emigration. More generally, factors that are strongly associated with a wish to leave can help set priorities for social policy.

In this paper we present a first of a kind systematic *inventory of surveys* that have collected data on migration aspirations. The inventory provides an overview of survey data and metadata from the past five decades to encourage further use and inform future research. (We are separately examining survey items and questionnaire design, and developing a *question bank* on migration aspirations; see Carling & Mjelva, 2021 for a preliminary version).

The study of migration aspirations touch upon several areas of migration research. This diversity is reflected in our references. In the inventory of surveys, we cite a total of 250 sources, of which 205 are journal articles. The articles are spread across 72 journals of which only 24 occur more than once. Table 1 lists them with their respective number of articles. Not surprisingly, the largest number of articles using data on migration aspirations are published in major migration journals. Other journals represent the fields of population studies, urban studies, development studies, rural studies and health policy.

**Table 1.** Journals with two or more articles cited in the inventory of surveys.

*Cities*	2	*Journal of Happiness Studies*	2
*Demography*	7	*Journal of International * *Migration and Integration*	5
*Demographic Research*	4	*Journal of Population* *Economics*	4
*Economic Development * *Quarterly*	2	*Journal of Rural Studies*	2
*Economic Thought*	2	*Population and Environment*	7
*Environment and * *Planning A*	4	*Population Research and * *Policy Review*	2
*Health Policy*	4	*Population Studies*	2
*Human Resources for * *Health*	7	*Population, Space and Place*	6
*International Migration*	12	*Rural Sociology*	4
*International Migration * *Review*	12	*Social Forces*	2
*IZA Journal of Migration*	3	*Sustainability*	4
*Journal of Development * *Economics*	5	*World Development*	2
*Journal of Ethnic and * *Migration Studies*	6		

In what follows, we present the inventory of surveys and its contents. In Section 2 we discuss the construction and organisation of the inventory, while Section 3 gives an overview of the metadata of the surveys. In our concluding remarks in Section 4, we discuss some recommendations and encouragements for survey reporting.

## 2 The inventory of surveys

In general, survey datasets exist in a variety of forms, with disparate degrees of public documentation and data availability. As a rule, they are not systematically indexed in databases in the way that, for instance, journal articles are. These factors make a review of surveys very different from a systematic review of literature.

We used publications as a gateway to establish an overview of surveys. Since we were looking for surveys that included items on migration aspirations, we conducted a search through Web of Science for literature that is survey-based and includes migration aspirations or related terms such as migration intentions or desires in the title or abstracts. ^1^ This search produced 287 hits, which were subsequently screened to identify publications that used relevant data. In addition, we searched the authors’ reference library of several thousand migration-related references, of which many relate specifically to migration aspirations. This library contains both articles, books, reports, and other publication types. Finally, the reference lists of selected articles were reviewed to identify additional potentially pertinent literature. Throughout the process, we did not discriminate by publication type or publication year. In total, we identified 289 publications that used survey data on migration aspirations, stemming from 212 surveys.

Inclusion in the inventory of surveys is contingent on three requirements. First, the survey must be of a quantitative nature, meaning that it must be structured with pre-formulated, standardised questions. However, no threshold concerning sample size was set to distinguish quantitative from qualitative surveys.

Second, the survey must contain at least one question inquiring about respondents’ migration aspirations. The question could concern residential mobility, domestic migration, international migration or migration at different geographical thresholds. It must, however, address the prospect of future migration, not respondents’ experience with migration in the past.

Third, it must be possible to obtain a minimum of information about the survey and survey instrument, beyond the fact that a survey exists. We did not have strict rules as to which metadata had to be available but needed *some* information, for example about the topic of the survey, the survey population, or geographic coverage, for it to be meaningful to include the survey in the inventory. Metadata on surveys is occasionally missing for data collection method (16%), sampling method (10%), data collection period (6%), survey design (6%) and sample size (1%). For the purpose of gaining an overview of relevant surveys, we included surveys with satisfactory survey-level information even if the information about specific survey items was faulty. The inventory of surveys contains the best information available in the referenced publications or survey documentation.

Each row in the inventory refers to one survey. Many surveys have rounds that vary in methodology, sample size, geographic coverage, or content of the survey instrument. Consequently, it is sometimes difficult to distinguish between rounds and independent surveys. This difficulty is compounded by the uneven availability of metadata, depending on how various rounds or parts of surveys have been used in publications. We have coded surveys as multi-round whenever they are described as such in the reference or survey documentation.

Each survey is given a numeric ID, assigned in the order of the first year of data collection, and then alphabetically by survey name among surveys with the same start year. If publications did not contain information about the data collection period, we assigned IDs with the assumption that data was collected three years before the publication year. In a few cases, information about additional rounds emerged during the review, with the result that not all IDs reflect the chronology of data collection.

Additionally, each survey in the inventory has a unique descriptive name. Some, like Afrobarometer or Gallup World Poll, have well-established official names. Others – especially one-off surveys carried out for a particular project – often lack a specific designation. In these cases, we have used the available information to formulate a name, such as ‘Migration Intentions among University Students in Slovakia’ or ‘Hubei Province Migration Survey’.

The inventory of surveys includes references to publications that have used each survey, typically the publication(s) through which each survey was identified in the first place. Hence, the list does not necessarily include all references that have used the survey but shows where we discovered the data. Some publications use several surveys and are therefore listed in several rows.

Although the search for surveys has been extensive and the list of surveys is long, we cannot assume that it is exhaustive. In particular, surveys carried out by international organizations, civil-society organizations, or private-sector actors are less likely to be used in scientific publications and could therefore more easily have been missed.

## 3 Overview of surveys

In what follows, we assess the geographic, temporal and population coverage, survey methods and data availability of the surveys. As we discuss them in this section, we refer to examples by their ID number, and refer to the underlying data for the full reference.

### 3.1 Geographic coverage

The inventory includes several measures of geographic coverage: geographic scale, number of countries covered, distribution across countries, and distribution across world regions.

We have classified the geographic scale of surveys as *subnational*, *national*, *multi-subnational*, *multinational*, and *diasporic*, as defined in Table 2. The multinational surveys cover between 2 and 155 countries, with a median of 6. Three surveys have a globally diverse coverage: the Gallup World Poll (76), which covers more than 150 countries, the International Social Survey Programme (18), which covers 42 countries and the Pew Global Attitudes Survey (51), which covers 25 countries. Almost all the remaining multinational surveys span a set of neighbouring countries within the same region.

**Table 2.** Distribution of surveys by geographic scale.

	Frequency		
Geographic scale	N	%	Description
Subnational	85	40	The survey covers one or more geographical areas within a country. Subnational surveys include those that cover only rural or urban populations, as well as surveys that use institutional samples that are not nationally representative.
National	76	36	The survey aims to be nationally representative of the survey population. National surveys include those that use institutional sampling to reach a nation-wide population, such as all medical doctors in the country.
Multinational	37	17	The survey covers more than one country and aims to be nationally representative within each country.
Multi-subnational	10	5	The survey includes more than one country but covers subnational populations within each country.
Diasporic	4	2	The survey covers migrants from the same country of origin who reside in various destination countries, sampled in diverse ways.

*Note: N* = 212.

Very few multinational surveys concentrate on migration issues. All those that cover more than seven countries are thematically broad surveys that have, at most, a handful of migration-related questions. By contrast, most of the multi-subnational surveys – which cover two to eight countries – are surveys that focus on migration or migration aspirations.

This variation in geographic scale cuts across variation in the *survey population*, which we discuss in Section 3.4. In other words, a national survey can target a highly specific population, such as British doctors in New Zealand (112) or Russian-origin immigrants in Israel (165). When we classify surveys as national or multinational – that is, with the aim of being nationally representative – we rely on descriptions in the cited publications or other survey documentation and have not evaluated the actual representativeness. However, surveys differ in the compromises they must make in the attempt to be representative at the national level.

The largest group of surveys are subnational, followed by the national ones. Only one in five cover more than one country. This distribution is unsurprising considering the lower resource requirements for subnational surveys. Some are products of graduate research, for instance.

The variation in geographic scale is partly linked to differences in the form of migration that is the focus of the survey. Many surveys explicitly address international migration, while others address internal migration or local residential mobility, and yet others do not discriminate between internal and international destinations.

The surveys cover countries from all parts of the world, though with clear imbalances. Classifying the geographical coverage of surveys is, in most cases, straightforward, though not always in surveys that cover migrant populations or that vary across rounds. ^2^

To map the distribution across regions we use the World Bank’s regional classification, presented in Table 3. The multiregional category describes surveys that include countries from more than one region, though they are, in some cases, a contiguous group of countries. The Afrobarometer (168), for instance, is multiregional because it spans the regions Sub-Saharan Africa and Middle East and North Africa.

**Table 3.** Regional classification.

	Frequency		
Abbreviation	N	%	Description
ECS	106	50	Europe and Central Asia
EAS	28	13	East Asia and Pacific
NAC	25	12	North America
SSF	14	7	Sub-Saharan Africa
LCN	12	6	Latin America and the Caribbean
MEA	10	5	Middle East and North Africa
SAS	3	1	South Asia
MR	14	7	Multiregional (including two or more of the regions listed above)

*Note:* Percentages do not add up to 100 due to rounding. *N* = 212.

Table 3 also displays the distribution of surveys across world regions. Europe and Central Asia top the list and strikingly account for half of all the surveys. At the bottom of the list is South Asia, which is represented by only three surveys: two from Pakistan and one from Afghanistan. South Asia, like other seemingly underrepresented regions, is also covered in multi-regional surveys.

**Figure 1.**
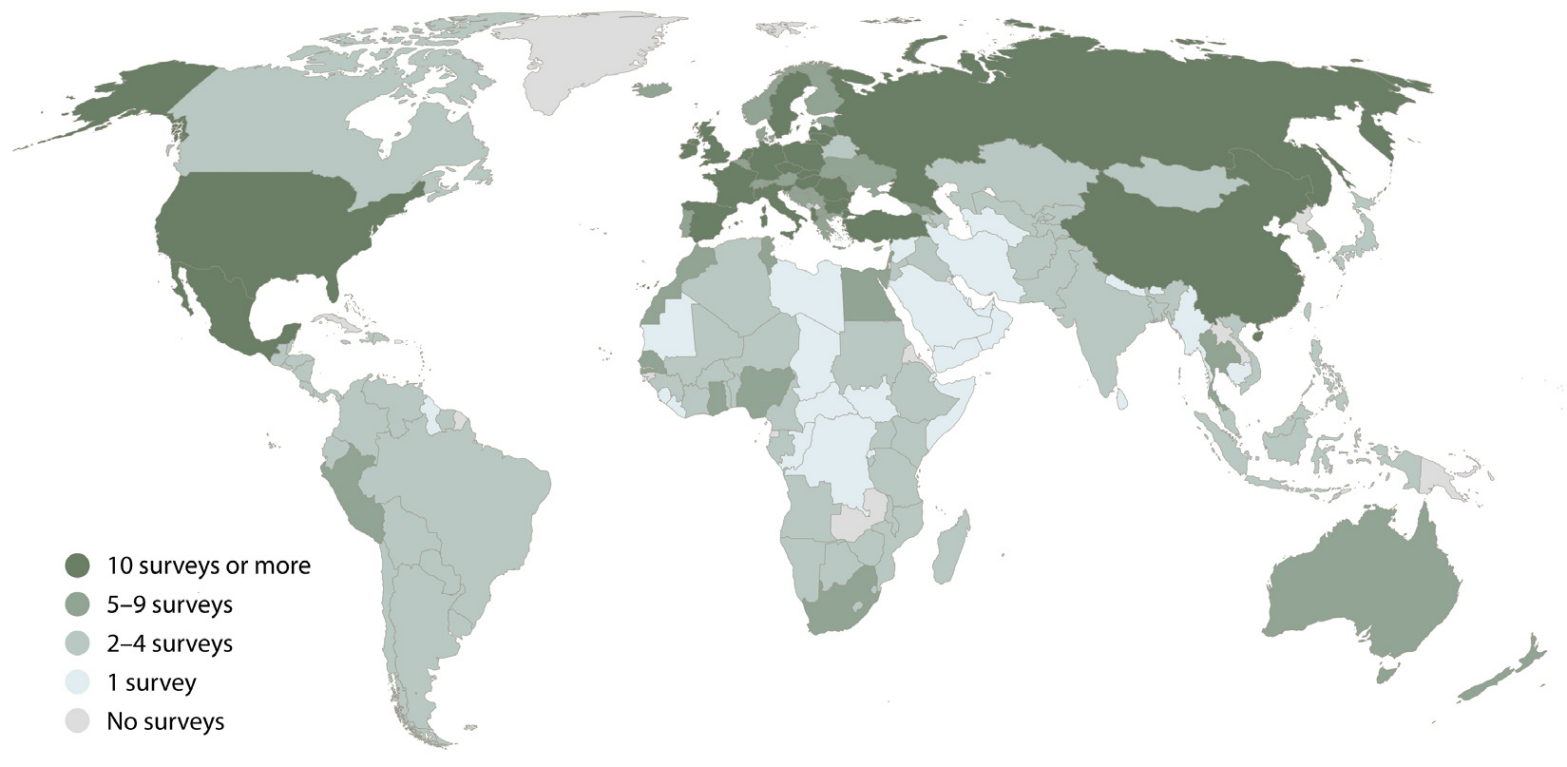
Frequency of coverage in surveys on migration aspirations.

Figure 1 offers a more fine-grained picture, displaying the country-level frequency of coverage. Of the 20 countries that appear in ten or more surveys, only three are non-European: the United States, Mexico, and China. In fact, the United States is the single most studied country, represented in 30 surveys. Next are the United Kingdom and Romania, with 20 surveys each. In addition to Romania, five other Central and Eastern European countries are among the ten most frequently represented (Hungary, Bulgaria, Poland, Czech Republic and Slovakia). This concentration of surveys partly reflects the policy-related interest in monitoring and forecasting East-West migration within Europe, triggered by the collapse of communist regimes, and later, by the expansion of the European Union. Yet, many of the surveys in Central and Eastern Europe are multinational or multi-subnational and tend to cover the same countries. For instance, Hungary and the Czech Republic appear together in 13 surveys. (The previously mentioned ambiguities about what should count as one survey also affect the numbers on country-level coverage. ^3^)

**Figure 2.**
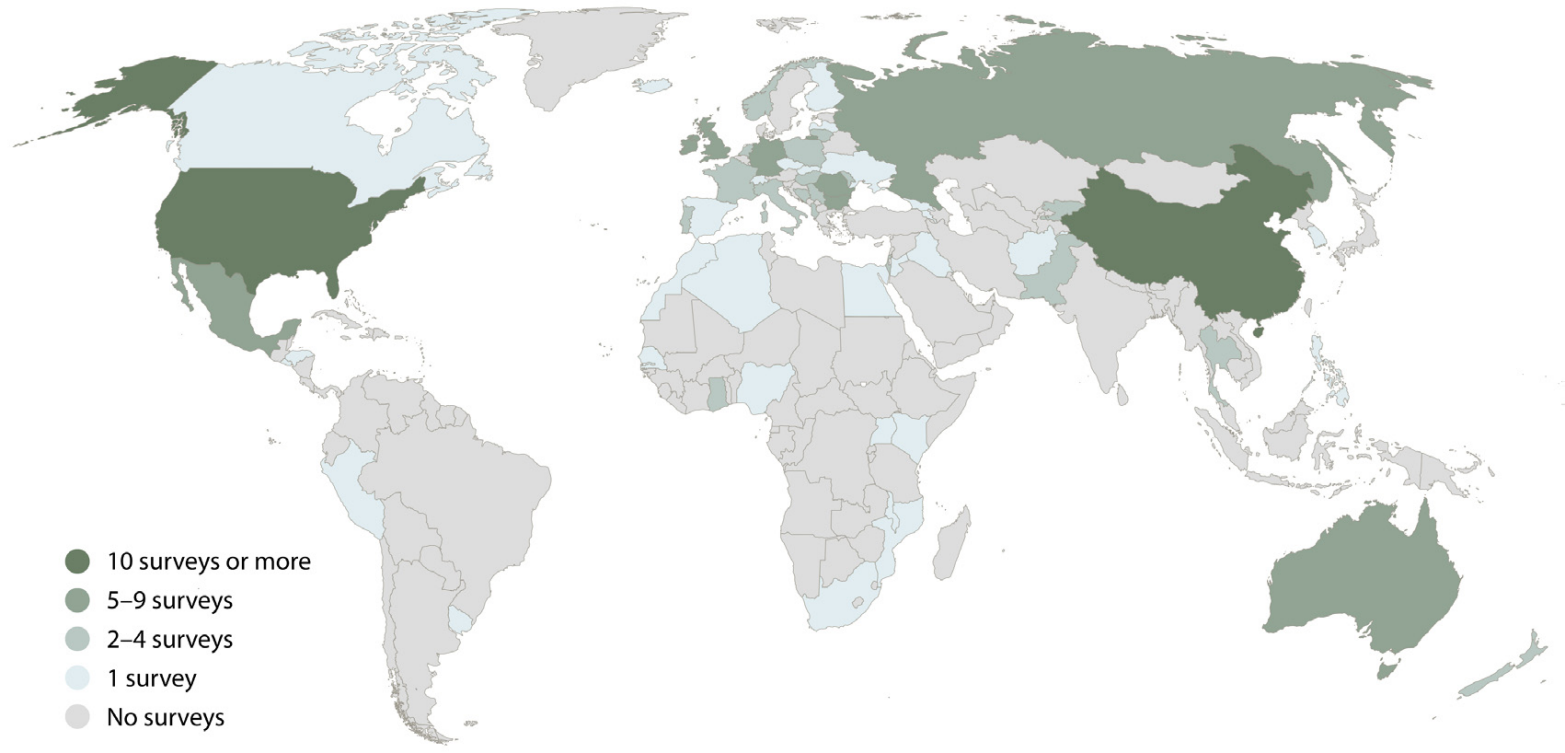
Frequency of coverage in national and subnational surveys on migration aspirations.

Beyond Europe, most countries are covered by fewer than five surveys. And very often, those surveys are standardized multi-national surveys. These surveys are immensely valuable for studying regional trends and making international comparisons but tend to be less attuned to context-specific dynamics. In Figure 2, we display the frequency to which each country appears in national and subnational surveys. Here we see that much of Latin America, Africa, the Middle East and Asia are not covered in any national or subnational survey. In contrast, the United States and China stand out with particularly many surveys of this type. Most of these surveys address internal migration. In the case of the United States, some focus on residential mobility in a single metropolitan area.

### 3.2 Temporal coverage

The inventory of surveys covers data that has been collected from the 1960s until 2020. Most collect data in a single round only, which could take anywhere from a few weeks to several years to complete. Other surveys collect data on the same population in multiple rounds – an aspect of survey design that we will discuss in Section 3.4. Data collection for such surveys can cover much longer periods, up to several decades. In the inventory of surveys, we have included the first and last year of data collection, to the best of our knowledge. ^4^

**Figure 3.**
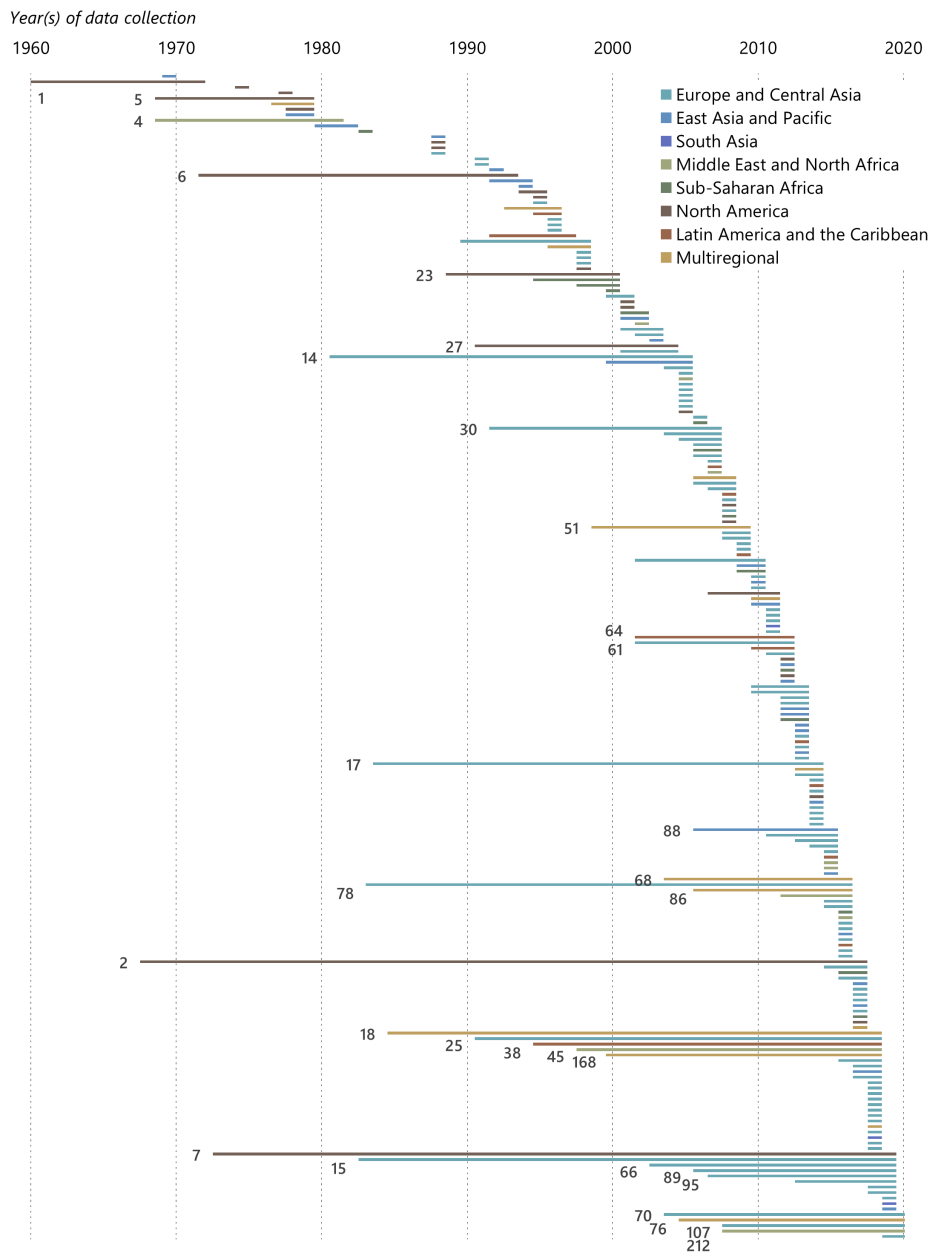
Data collection periods by region. *Note:* Only surveys with a data collection period of at least ten years are labelled. Where the data collection period is not reported in the reference, we have estimated it by assuming that data was collected three years before the publication year of the reference. See the underlying data for details on each survey.

Figure 3 displays the data collection period for each survey. The period is the interval between the first and last year of data collection, regardless of the frequency of data collection in between. In multi-round surveys, data might be collected annually during this time span, or less often, or at less regular intervals.

All surveys covering a time span of ten years or more are labelled in the figure. Two thirds of these long-running surveys cover either Europe and Central Asia or North America. The two longest-running surveys are the Panel Study of Income Dynamics (2) and the American Housing Survey (7), both of which are national surveys in the United States.

### 3.3 Survey population

Most of the surveys cover general populations, but almost as many are targeting specific groups. Each survey draws a sample from a pre-defined population with certain characteristics, and the difference in population is a key form of variation between the surveys. Table 4 displays the distribution of surveys across population categories. Just over half of the surveys cover the general population, though some are limited to specific age groups.

**Table 4.** Distribution of surveys by population category.

Population category	Frequency		
	N	%	Description
General population	115	54	All residents in the geographic area covered. In some cases, data is obtained from heads of households, but also cover other household members.
Students	41	19	Pupils or students at any level of education, from high school to graduate programs. Populations are often restricted to specific grades or disciplines. Surveys of recent graduates are included in this category.
Migrant population	18	8	Migrants, and sometimes children of migrants, or others with a migrant background. The populations may be defined by either internal or international migration and may cover migrants from a single origin country or of multiple origins.
Health workers	12	6	Professionals within the health sector, primarily physicians and nurses.
Health worker migrants	5	2	Defined by the overlap of the ‘migrant population’ and ‘health workers’ categories (all surveys in this category cover physicians who live in a country other than their country of citizenship or training).
Health sciences students	4	2	University students in health sciences (all four surveys in this category cover medical students).
Other	17	8	Populations not covered by any of the above groups. Examples include married women, employees at a particular company, or individuals who identify as LGBT.

*Note:* Percentages do not add up to 100 due to rounding. N = 212.

The most common specific population category is *students*. The prominence of students has several possible explanations. Some surveys are linked to the growing interest in international student mobility, especially in Europe. Other surveys among students may be motivated by concerns about human capital losses (so-called ‘brain drain’, see for instance Gibson & McKenzie, 2011 for more information about the concept). Finally, student populations can be appealing for logistical and financial reasons when sampling and recruitment can be organized through schools, universities or associations. Migration aspirations is particularly prominent among youth and young adults, who, in many countries, are likely to be students. Consequently, a sample of students could reflect an interest in the age group, combined with logistical sampling considerations, rather than a specific emphasis on respondents being students.

A second prominent category includes *migrant populations*, which in some surveys include children of migrants (78, 90, 127). It is common in surveys of migrants to include questions about plans or wishes for return or onward migration, which can be seen as a particular form of migration aspirations.

The third most frequent category is *health workers*, often defined as physicians or nurses. The emigration of health workers is a major policy concern in many countries, and understanding their migration aspirations, and the underlying motivations can therefore be important. The majority of these surveys were undertaken in European countries with significant out-migration, such as Ireland, Poland and Portugal.

The prominence of student, migrant, and health worker populations was reflected in several overlaps between these categories. We have therefore singled out health worker migrants and health sciences students as population categories.

In addition to differences in population type, the surveys differ considerably in the age range of their samples – as well as in the level of detail on age that is provided in publications. We have used the available information to classify the surveys into three broad groups ( Table 5). The most important difference between surveys is the upper bound of the age range, which we use as the criteria for distinguishing between *adult*, *young adult*, and *youth* samples. The lower bound of the age range also varies, though not always in expected ways. For instance, some surveys covering the adult population include individuals down to the age of 14.

**Table 5.** Distribution of surveys by age range.

	Frequency		
Age range	N	%	Description
Adults	147	69	Surveys where the upper bound is larger or equal to 40 years
Young adults	48	23	Surveys where the upper bound is equal to or between 25 and 39 years
Youth	17	8	Surveys where the upper bound is lower than 25 years

*Note: N* = 212.

Almost one third of the surveys are limited to youth or young adults. Many of these surveys cover students, and some focus on migration aspirations of youth and young adults from rural districts. Migration aspirations decline with age ( Aslany *et al*., 2021), and surveys that specifically address this topic can therefore benefit from concentrating available resources on a younger sample.

### 3.4 Survey methodology

In this section we address four aspects of survey methodology: (1) the overall design in terms of data collection in one or more rounds over time, (2) the method of sampling respondents, (3) the size of the sample, and (4) the form in which respondents provide information.

A fundamental aspect of survey design is the way of which data is collected over time. There can be one or more rounds of data collection, and if there are several rounds, respondents can be the same or be replaced in each round. For simplicity, we use three main categories, presented in Table 6: *single-round* surveys, *multi-round* surveys, and *longitudinal* surveys. In addition, a few surveys have a mixed design with consecutive, disconnected panels. For about a dozen surveys, there is insufficient information about the survey design to allow for categorization.

**Table 6.** Survey design.

	Frequency		
Survey design	N	%	Description
Single-round	132	62	The survey is conducted once, as a cross-sectional survey, with one instance of data collection from each respondent.
Multi-round	34	16	The survey is conducted several times, as a series of cross-sectional rounds with new samples. The population is the same, or similar across rounds, but each respondent provides information only once.
Longitudinal	31	15	The survey is conducted in two or more rounds with the same panel of respondents. Each respondent provides information at least twice.
Mixed	2	1	The survey combines aspects of multi-round and longitudinal designs by drawing two or more consecutive panels.
Missing	13	6	The survey design was not possible to verify based on the information provided in publications or survey documentation.

*Note: N* = 212.

Overall, about a third of the surveys have multi-round or longitudinal designs, allowing for analyses of trends or dynamics over time. These are primarily surveys of the general population. There is only one such survey among the 21 that cover workers and students in the health sector.

Comparability and continuity across multiple rounds of a survey vary. First, the selection of countries or other aspects of the target population could differ. Afrobarometer (168) for instance, has collected data in multiple rounds since 1999 and covered a total of 40 countries, but the first round covered only 12. Similarly, the survey Living Conditions among Immigrants in Norway (78) has been carried out roughly every decade, covering a selection of immigrant groups that has changed from round to round. Second, questions about migration aspirations are not necessarily included, or formulated in the same way, in every round.

Survey respondents can be sampled in diverse ways, which we have classified in three broad categories ( Table 7). *Random or quasi-random* sampling methods seek to give each individual in the population the same probability of being included in the sample. In practice, randomness is a matter of degree, depending on compromises that are made in the design and execution of the survey. At the same time, standards for describing a survey as ‘random’ vary across research communities. We therefore use a broad category that also includes quasi-random designs in which the deviations from randomness are explicit. Two thirds of the surveys in the inventory of surveys fall into this category.

**Table 7.** Sampling method.

Sampling method	Frequency		
	N	%	Description
Random or quasi- random	142	67	The survey uses sampling that approximates the ideal that each individual in the population has the same probability of being included in the sample.
Institutional sampling	36	17	The survey recruits respondents via institutional affiliation, sometimes with a gross sample that is the same as the population.
Non-random	13	6	The survey samples respondents in ways that cannot be described as random, for instance through respondent-to-respondent referrals (snowball sampling).
Missing	21	10	The sampling method was not possible to verify based on the information provided in publications or survey documentation

*Note: N* = 212.

The second method is what we have called *institutional* sampling, in which individuals are sampled on the basis of an institutional affiliation. Examples include students at a university, employees of a company, members of an association, and similarly aggregated samples from multiple institutions of the same type. In some cases, the gross sample is the same as the population. For instance, if the population is defined as all medical students in a country, the entire population might be contacted *via* their universities, and the difference between the population and the sample would be accounted for by non-response. Overall, 17% of the surveys used institutional sampling. This proportion was twice as high in surveys of students and represented the vast majority of surveys on health workers.

Third, several surveys used explicitly *non-random* sampling methods. These include snowball sampling, by which respondents refer to other potential respondents. Surveys that authors describe as non-probabilistic have been placed in this category. Non-random sampling was used in only 6% of the surveys.

Basic information about sampling methods was missing for 10% of the surveys. In most cases, the publications or documentation mentioned sampling but described it too briefly or superficially for classification. Without proper information about sampling method, it is impossible to assess the representativity of surveys.

The sample size of the surveys varies by a factor of 4,000 from the smallest (40 respondents) to the largest (161,000 respondents). For multi-round surveys we have recorded the sample size as reported in the publications that are cited as sources for each survey. If information is available for more than one round, we have used the largest sample size.

**Figure 4.**
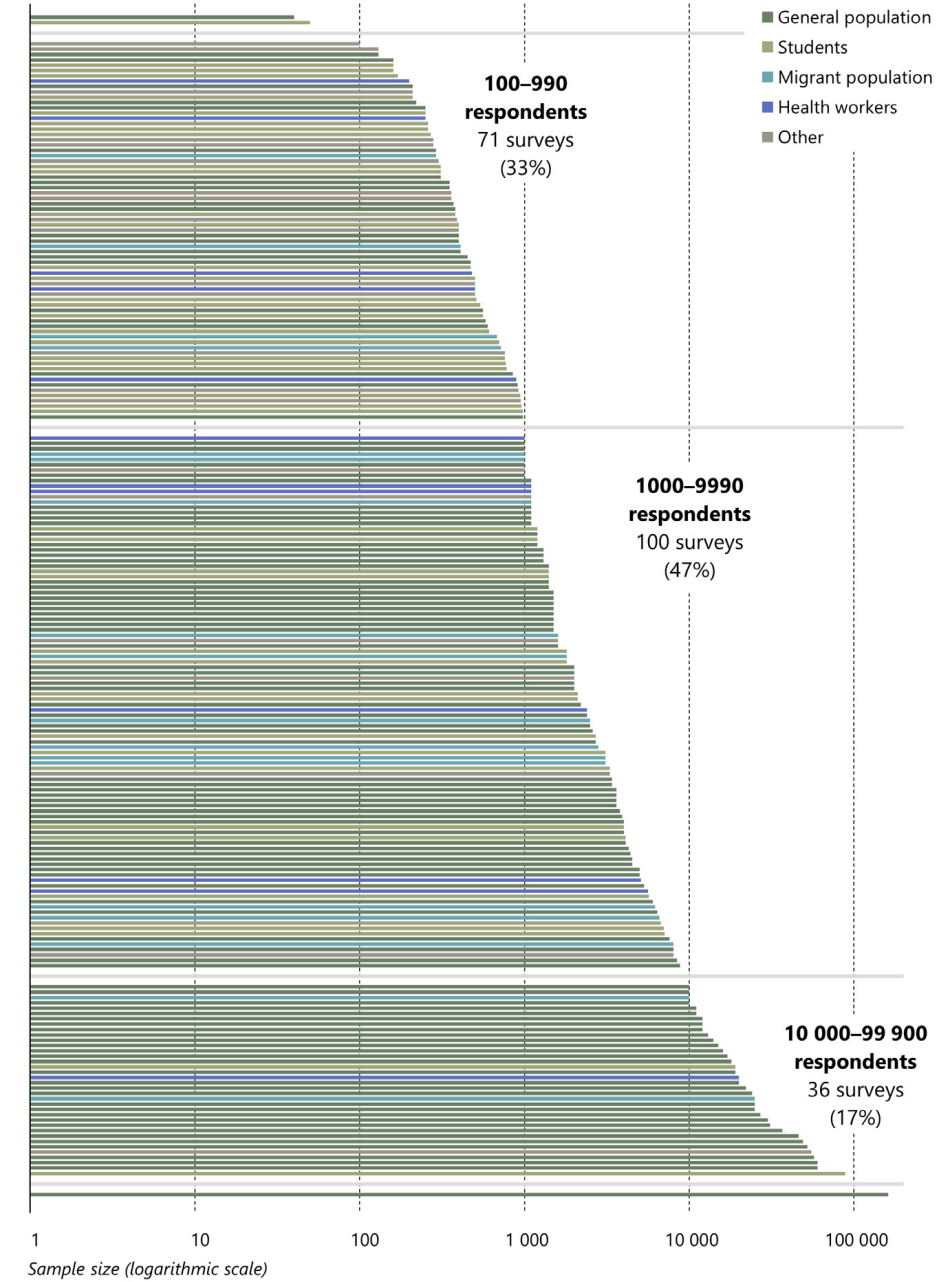
Distribution of surveys by sample size and population. *Note:* In the classification of survey populations ‘students’ include health sciences students and ‘other’ include health worker migrants. N = 212.

Figure 4 displays the distribution of surveys by sample size and population category. Only one survey (76, the Gallup World Poll) has a sample of more than 100,000 respondents, while another 36 surveys have samples of 10,000 respondents or more. As can be seen in Figure 4, surveys of the general population dominate among these large surveys, although there are surveys of every other main population category with samples of at least 10,000 respondents. Several of the largest surveys are multinational and their samples for each country are not necessarily large.

Survey data can be collected in a number of ways that have diverse benefits and disadvantages, for instance in terms of costs and accuracy. The distinction that matters most for data content and reliability is whether the data was collected in conversation with an enumerator or entered directly by the respondent in a questionnaire or on a screen. We have classified the surveys based on this distinction and labelled the data collection method as either *interview* or *self-administered* ( Table 8).

**Table 8.** Data collection method.

Data collection method	Frequency		
	N	%	Description
Interview	114	54	Data was collected through interviews, which were either face- to-face or conducted by phone, and either computer-assisted or paper-based.
Self-administered	62	29	Data was entered directly by respondents, either electronically or on a paper questionnaire.
Mixed	3	1	Data was collected by a combination of interviews and self- administered responses.
Missing	33	16	The data collection method was not possible to verify based on the information provided in publications or survey documentation

*Note: N* = 212.

The majority of surveys collect data by means of interviews, either in person or by phone. Compared to self-administered data collection, interviews provide greater opportunities for quality assurance, though this potential depends on interviewer skills and training. The feasibility of self-administered data collection depends, among other things, on the qualifications of respondents and the complexity of the survey.

Even with a simple distinction between two broadly defined data collection methods, 33 surveys (16%) were not possible to classify based on the available information. A few surveys combined the two formats. In some cases, publications stated that data was collected by means of questionnaires but failed to specify whether they were completed by interviewers or respondents.

### 3.5 Survey items on migration aspirations

Survey items that enquire about migration aspirations can be broken down in terms of their *mindset* and a *ction*. Here, we discuss the *nature of the mindset* of the items. For more information about the composition of survey items on migration aspirations, see Carling (2019) and Carling & Mjelva (2021).

The nature of the mindset can broadly be described as a person’s thoughts and feelings about the prospect of migration. Carling (2019) identified eight types of mindsets: consideration, preference, willingness, necessity, planning, intention, expectation, and likelihood. A description of these mindsets is found in Table 9, while Table 10 shows the distribution of the mindsets across the surveys. A list of the types of mindsets used in each survey is found in the underlying data.

**Table 9.** Definitions of the eight categories of mindset.

Nature of the mindset	Definition (with the action defined as ‘migration’)
Consideration	The act of reflecting on the feasibility or desirability of migration
Preference	The evaluative conclusion that migrating would be preferable to staying
Willingness	The preparedness to migrate despite assumed disadvantage or hardship
Necessity	The assessment that migration is the only option
Planning	The preparation of a course of action towards migration
Intention	The will or commitment to pursue a course of action towards migration
Expectation	The belief that migration will most probably take place
Likelihood	The assessment of the probability that migration will take place

*Note:* Dashed lines indicate closely related mindsets.

**Table 10.** Specifications of the nature of the
mindset.

Nature of the mindset	Frequency		
	N	%	Example
Consideration	28	13	Have you, in recent times, seriously considered moving abroad for an extended period or forever? (17)
Preference	34	16	Would you like to move from your current location to a different place at some point within the next 10 years? (149)
Willingness	16	8	How willing would you be to live in another [current world region] country where the language is different from your mother tongue? (56)
Necessity	0	0	I feel that I’m going to have to migrate to [main destination country] when I graduate in order to ﬁnd a job to support myself or my family (110)
Planning	12	6	Are you planning to move permanently to another country in the next 12 months, or not? (76)
Intention	33	16	Do you have any intention of going to live or work in another country in the next three years? (68)
Expectation	15	7	Do you think you will ever move back to your country of origin, or that of your parents, to live there permanently? (78)
Likelihood	11	5	How likely is it that you might move out of the present community in the next three years? (8)
Multiple	36	17	How likely is it that you will move to [main destination world region]? (24) AND Have you considered working in [main destination world region]? (24)
Other	5	2	Are you currently planning or considering moving to another country? (132)
Missing	22	10	

*Note:* Two surveys included an item with a *necessity* mindset. Both surveys had multiple survey items on migration aspirations, which means that the nature of the mindset for these surveys are coded as *multiple*. *N* = 212.

Some surveys have more than one question enquiring about migration aspirations, and thus account for more than one mindset. These are marked as ‘Multiple’ in Table 10. Moreover, 2% of the surveys had items that could not be classified according to our framework because they combine several types of mindsets in the same question and/or response categories. These are labelled ‘Other’ in Table 10. Lastly, 10% of the surveys are coded as missing. ^5^

*Consideration* generally refers to cognitive behaviour in the past, simply differentiating between those who have given migration some thought and those who have not. *Preference*, *willingness* and *necessity* reflect some form of comparison between the expected outcomes of leaving and staying. If migration is seen to be ‘necessary’ it could be interpreted as an extreme version of preference in which the option of staying is so firmly rejected that it is considered impossible. *Intention* and *planning* both represent the next step, from evaluation towards action, and therefore appear to be more tangible than preferences, for instance ( Tjaden *et al.*, 2019; van Dalen & Henkens, 2008), though these concepts are marred by other shortcomings ( Carling & Mjelva, 2021). Finally, *expectation* and *likelihood* stand out because they concern beliefs about future outcomes. Regardless of whether individuals would prefer to migrate and intend to do so, they could see it as most likely that they end up staying. The most widely used mindsets are *preference* and *intention*.

### 3.6 Summary of survey characteristics

**Figure 5.**
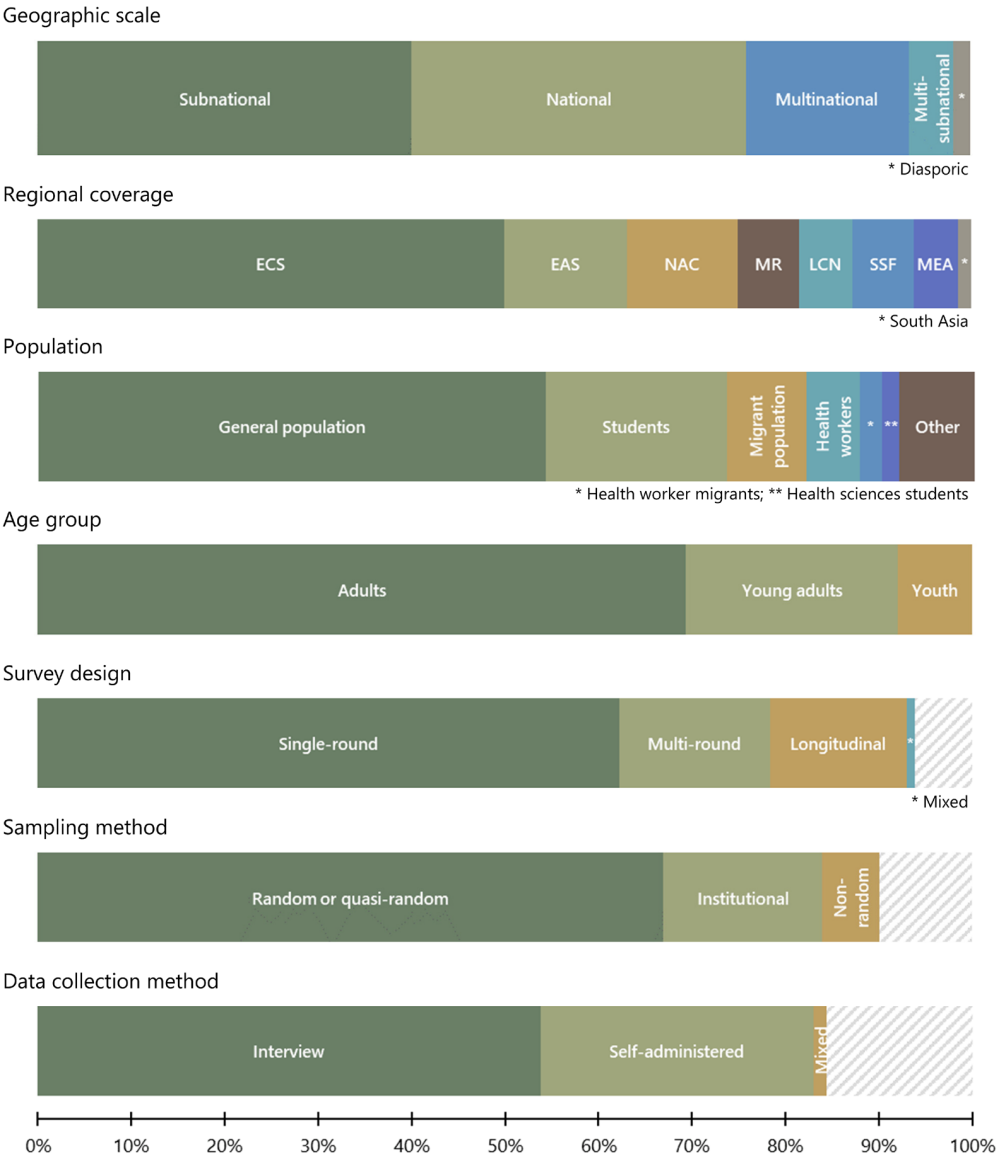
Overview of survey characteristics. *Note:* Grey hatching represents missing data. *N* = 212.

We have so far addressed key characteristics one by one and presented frequency distributions across categories in a series of tables. Figure 5 provides a visual display of these frequency distributions. For each characteristic, the most common category accounts for more than half of the surveys. In other words, a ‘typical’ survey that combines all the modal categories would be a single-round sub-national survey in Europe or Central Asia that covers the general population of adults with random or semi-random sampling and collects data through interviews. However, only five surveys (81, 157, 188, 200, 210) share this combination of characteristics.

**Figure 6.**
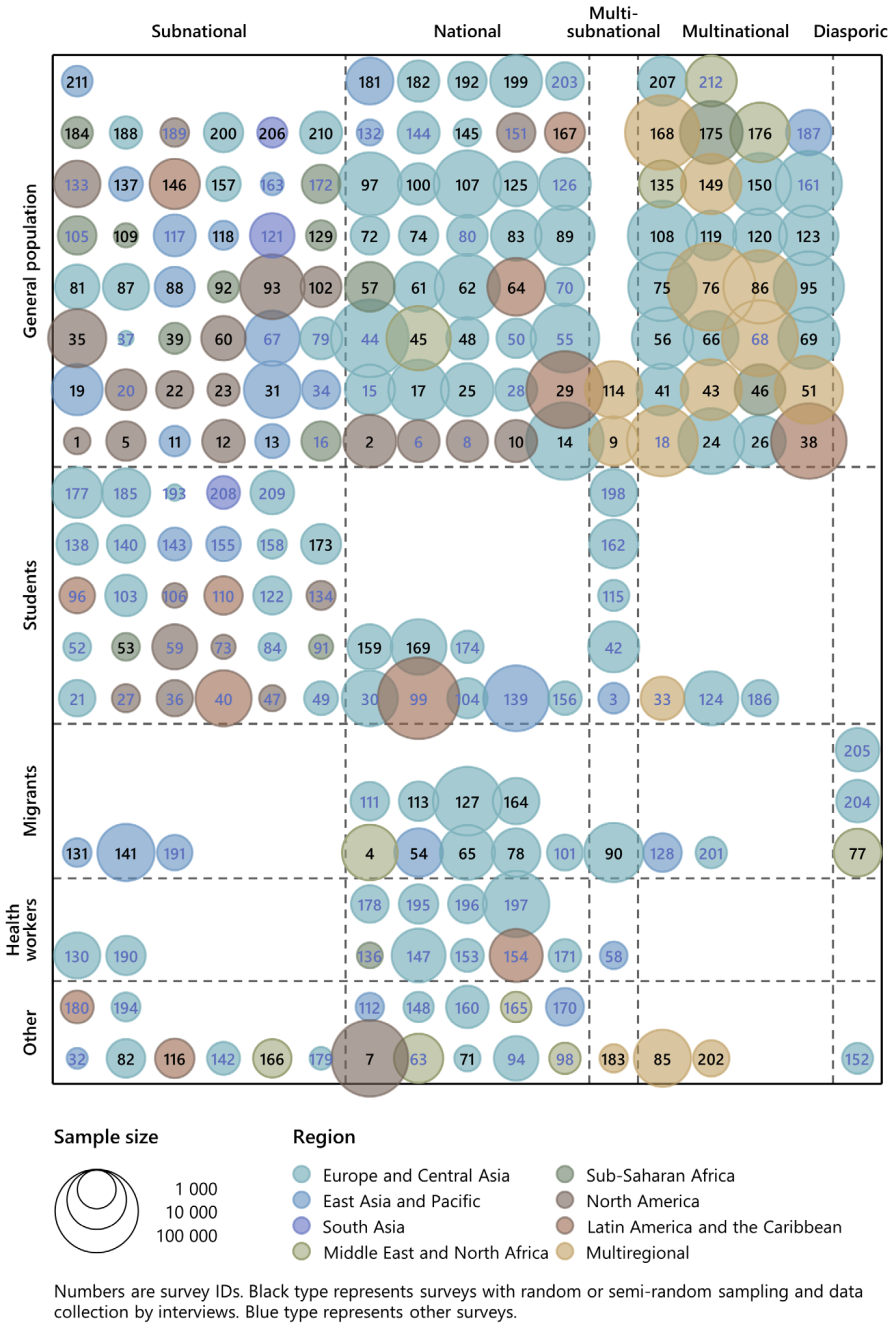
Surveys by survey population and geographic scale. *Note:* Numbers are survey IDs. Black type represents surveys with random or semi-random sampling and data collection by interviews. Blue type represents other surveys. In the classification of survey populations ‘students’ include health sciences students and ‘other’ include health worker migrants.

To explore variation across characteristics, we present Figure 6, which displays all 212 surveys by geographic scale and population, differentiated by regional coverage. The figure also identifies surveys that used random or quasi-random sampling methods and gathered responses through interviews rather than self-administration. Table 11 lists the surveys in the same order as the figure for easy reference.

**Table 11.** Surveys listed in the order of display in Figure 6.

Subnational | General population
211. Moving Intentions among Residents in Renovated Chinese Historical Blocks
210. Migration Intentions Survey in Tirana, Albania
206. Survey of Youth Urban Migration Intentions in Khushab, Pakistan
200. PAPI Survey on Life Quality in Lublin*
189. Sense of Community and Migration Intentions of Rural Youth in Ohio
188. Place Attachment among Residents of Belgrade
184. Household Survey of the Upper River Region in the Gambia
172. Migration Intention Survey of Slum Dwellers in Lagos, Nigeria
163. Poverty, Urban Attraction and Migration in Northern China
157. Survey of Personal Plans for Migration in the City of Stabropol
146. National Adolescence and Youth Survey*
137. Survey of households located in Areas at risk for Tsunami
133. Quality of Life of Residents in South Dakota
129. Household Survey in Volta River Delta in Ghana
121. Afghanistan Household Survey
118. Questionnaire of households in Minqin County
117. Migration Aspirations in Abkhazia and South Ossetia
109. Malawi Wet-Season Migration Survey
105. Survey from two provinces in the South of Mozambique
102. Rural Utah Community Study*
93. Carsey Institute’s Community and Environment in Rural America*
92. Willingness to Migrate Illegally in Dakar, Senegal
88. Rural Household Survey in Hubei Province China
87. Magdeburg and Freiburg Survey
81. Resettlement Pattern in the North Caucasus
79. Migration Intentions in Kyrgyzstan after the Tulip Revolution
67. Youth Intentions to Stay in Home Communities
60. The Northern Plains Survey*
39. Observatório de Migracões e Emprego*
37. Experimental Study of Portuguese Teenagers and their Migration Aspirations
35. Project on Human Development in Chicago Neighborhood*
34. Household survey of Tongans and Western Samoans in Sydney
31. National Migration Survey of Thailand*
23. Iowa Youth and Family Project*
22. Utah Migration Telephone Survey
20. Mobility Expectations among Residents in Phoenix
19. Hubei Province Migration Survey
16. Survey in rural Kenya
13. The Philippine Migration Study*
12. Residents of Seattle Mobility Survey
11. Northeast Thailand Village Survey
5. Mobility and Residential Satisfaction Survey Rhode Island
1. Migration Survey Durham, North Carolina
Subnational | Students
209. Migration Intentions of Romanian Engineering Students
208. Migration Aspirations among Students at the University of Lahore
193. Career Orientation among Students at a Boarding School
185. Intention to Migrate to Western Europe among Students in Romania
177. Study of the Factors that Cause Young Specialists to Leave the Russian Arctic
173. Migration Intentions after Graduation among Students in Romania
158. Survey of Students in Barnaul from Rural Parental Municipalities in Altai Krai
155. Perceptions of English-Medium Instruction and Migration Intentions in Hong Kong
143. Migration Intentions among Students in Nanjing
140. Emigration Intentions of Future Romanian Physicians
138. Survey of Spanish Students Studying German in Spain
134. Rural Youth Community Survey*
122. Belgrade Students of Medical Faculty Survey
110. Migration Intentions among Mexican Adolescents
106. Survey of College Students in Appalachia, Kentucky
103. Study abroad survey of students in Brighton, Sussex, Leicester and Leicestershire in England
96. Mexican High School Students Survey
91. Uganda Nursing School Study
84. CYFLO Project Survey
73. Chinese Students in Canada Survey
59. Migration Patterns of Graduate Students in Pittsburgh
53. Student Survey Cape Verde
52. Identity and Migration Intentions Student Survey University of Sussex
49. Survey of Students in two larger cities in Bulgaria
47. Migration Intentions among Master of Business Administration Graduates
40. School Student Migration Aspirations Mexico
36. Alaska Youth Studies
27. Pittsburgh Student Survey
21. Survey of Shetland and Orkney High School Students
Subnational | Migrants
191. Survey of Rural-Urban Migrants in Beijing and Jinzhou
141. Intended Place of Residence in Old Age of Internal Migrants in Shanghai
131. Migration Intentions of Resettled People in West China
Subnational | Health workers
190. Survey of physicians, nurses, residents, and medical students in Lithuania
130. Migration Intentions among Physicians in Germany
Subnational | Other
194. Intention to Migrate among Employees in Kosovo
180. Youth Outreach Centers in El Salvador
179. Youth Entrepreneurship and Emigration Intentions
166. Survey of Potential Refugees in Baghdad
142. Migration and Unemployment in Ukraine
116. Migrant Border Crossing Survey*
82. Survey of Married Women in Rural Armenia
32. Workers Mobility Intentions Hong Kong
National | General population
203. Survey of Migration Intentions of Employed Romanian Citizens
199. National Youth Survey in Bosnia and Herzegovina*
192. Tárki Omnibus Survey*
182. European Values Study Albania*
181. Community Wellbeing National Survey*
167. Willingness to Conduct Undocumented Migration in Honduras
151. Internet Survey on Migration Aspirations in a Global North Sending Country
145. Migration Survey Moldova
144. Migration plans in Hungary among the 18-40 aged population*
132. New Zealand Mobility Intentions
126. Online Survey of UK Population on Past and Future Migration
125. Nationally Representative Household Survey in Kyrgyzstan
107. Trajectoires et Origines*
100. Kosovo Emigration Intentions Survey
97. Norwegian Generations and Gender Survey*
89. The Panel Study Labour Market and Social Security*
83. CBSAXA Survey*
80. NIDI emigration survey*
74. Emigration Intentions of Latvians
72. Bulgaria Household Survey
70. Italy Labour Force Survey*
64. Mexican Family Life Survey*
62. Early Warning System Project
61. Albanian Living Standards Measurement Survey*
57. HSRC Migration Survey *
55. Bulgaria Census
50. Survey on Economic Expectations and Attitudes
48. Migration Intentions in Albania
45. Egypt Labor Market Panel Survey*
44. The Spanish Labour Force Survey*
29. Encuesta Nacional de Dinámica Demográfica*
28. Social Atlas of Romania
25. British Household Panel Survey*
17. German Socio-Economic Panel*
15. British Social Attitudes Survey*
14. Housing Demand Survey*
10. Quality of Employment Survey*
8. NORC Amalgam Survey*
6. Preference and Residence
2. Panel Study of Income Dynamics*
National | Students
174. Migration Intentions among University Students in Slovakia
169. Assessment of Migration Potential of Graduate Students of Higher Educational Institutions of CIS Countries*
159. Transition from School to Work survey*
156. Survey of Agricultural Students in Bulgaria
139. Chinese Education Panel Study*
104. Study of medical students in five universities in Poland
99. Exámenes de la Calidad y el Logro Educactivos*
30. Icelandic Youth Migration Intentions Surveys
National | Migrants
164. Settlement or Mobility?*
127. Social Condition and Integration of Foreign Citizens*
113. Causes and Consequences of Early Social and Cultural Integration Processes among Recent Immigrants to Europe*
111. Survey of Estonian Origin Migrants in Finland
101. Riinvest Migrant’s Survey*
78. Living Conditions among Immigrants in Norway*
65. Passage à la Retraite des Immigrés*
54. The Longitudinal Survey of Immigrants to Australia*
4. Immigration Absorption Survey*
National | Health workers
197. Migration Intentions among Health Professionals in Portugal
196. Migration Intentions among Doctors in Hospitals in Poland
195. Migration Intention Survey of Junior Hospital Doctors in Ireland
178. Your Training Counts Survey*
171. MadTreck Study*
154. National Survey of Health Users*
153. Migration Intentions among Medical Residents in Portugal
147. Online Survey of Physicians and Dentists Working in Hungary
136. Study of actively practicing physicians in Ghana
National | Other
170. Hong Kong Lesbian, Gay, and Bisexual Consideration of Emigration
165. Survey of Immigrants in Israel with Russian Background
160. Duration of stay for Migrant Physicians in Germany
148. Survey of Foreign Doctors Working in Ireland
112. UK Doctors in New Zealand
98. Survey of Egyptian Physicians Residing in Jordan
94. Dutch Potential Workforce Survey
71. Potential Migrants from Expat Fair Netherlands Survey
63. L’enquête algérienne sur la Santé de la Famille*
7. American Housing Survey*
Multi-subnational | General population
114. EUMAGINE*
9. Mobility Intentions in Thailand, Egypt and Colombia
Multi-subnational | Students
198. Migration Intentions among Students in Romania and Moldova
162. Future Migration Plans of University Graduates in the Netherlands, Germany and Belgium
115. Ireland Student Survey
42. Cross-Cultural Study of Rural Youth’s Migration Intentions
3. Australasian Undergraduate Students Survey
Multi-subnational | Migrants
90. TIES Project Survey*
Multi-subnational | Health workers
58. Migration of doctors and nurses from South Pacific Island Nations
Multi-subnational | Other
183. Household Survey in the Marshall Islands
Multinational | General population
212. Arab Youth Survey
207. YOUMIG*
187. Migration Survey from Malaysia, Indonesia, and the Philippines
176. SAHWA Youth Survey*
175. Nationwide Migration Surveys in West Africa
168. Afrobarometer*
161. European Young Adult Online Survey
150. Eurobarometer (Flash 395)*
149. Young Lives Project*
135. School-to-Work Transition Survey*
123. Friedrich-Ebert-Stiftung Youth Studies in East Europe*
120. Willingness to Migrate or Commute in Czech Republic, Slovakia and Hungary
119. The Effects of Migration on Children and the Elderly Left Behind in Moldova and Georgia*
108. Eurobarometer (Special 337)*
95. Eurobarometer*
86. Life in Transition Survey*
76. Gallup World Poll*
75. Eurobarometer (Mobility Survey)*
69. Austrian Labor Market Monitoring Survey*
68. AmericasBarometer*
66. Caucasus Barometer*
56. Eurobarometer (Candidate Countries)*
51. Pew Global Attitudes Survey*
46. Migration and Health Survey
43. Multicountry Migration Study
41. Central-Eastern Europe Migration Potential Survey
38. Latinobarómetro*
26. Migration Intentions Survey in Former Soviet Block Countries
24. Eurobarometer (Central and Eastern Europe)*
18. International Social Survey Programme*
Multinational | Students
186. Migration Intentions among Master Students in Portugal and the Netherlands
124. Intra-European Student Mobility Survey
33. Migration desires among college students in four countries
Multinational | Migrants
201. Past and Future Plans of Migrants in five EU countries
128. Asian International Students in South Korea, Japan and China
Multinational | Other
202. Return Aspirations among Syrian Refugees in Turkey and Lebanon
85. ETF Potential Migration Survey
Diasporic | Migrants
205. Survey of Romanians Living Abroad
204. Survey of Romanian Migrants in Western Europe
77. Les Marocains Résidant à l’Étranger*
Diasporic| Health workers
152. Migration Intentions among Irish Medical Professionals Abroad

*Note:* Asterisks indicate official names. See the underlying data for additional information.

### 3.7 Data availability in sampled surveys

It is increasingly the norm to make research data publicly available, though this is far from universally the case. We have coded the availability of survey data based on information in the publications or other documentation, in two broad categories. The survey data is deemed *available* if, according to the publications, it can either be downloaded or obtained upon request, with or without a fee, and with or without specific restrictions or conditions. Data from the remaining surveys is deemed *not available* (the two classifications are coded as ‘yes’ and ‘no’ in the stated data availability column of the underlying data). Overall, data was reported to be available for 25% of the surveys.

The data availability information is an indication, but no guarantee either way. When a publication from several decades ago states that data is available upon request, it might not be possible to obtain today. Likewise, if publications do not state explicitly that data is available, it could, nevertheless, be possible to obtain upon request.

**Figure 7.**
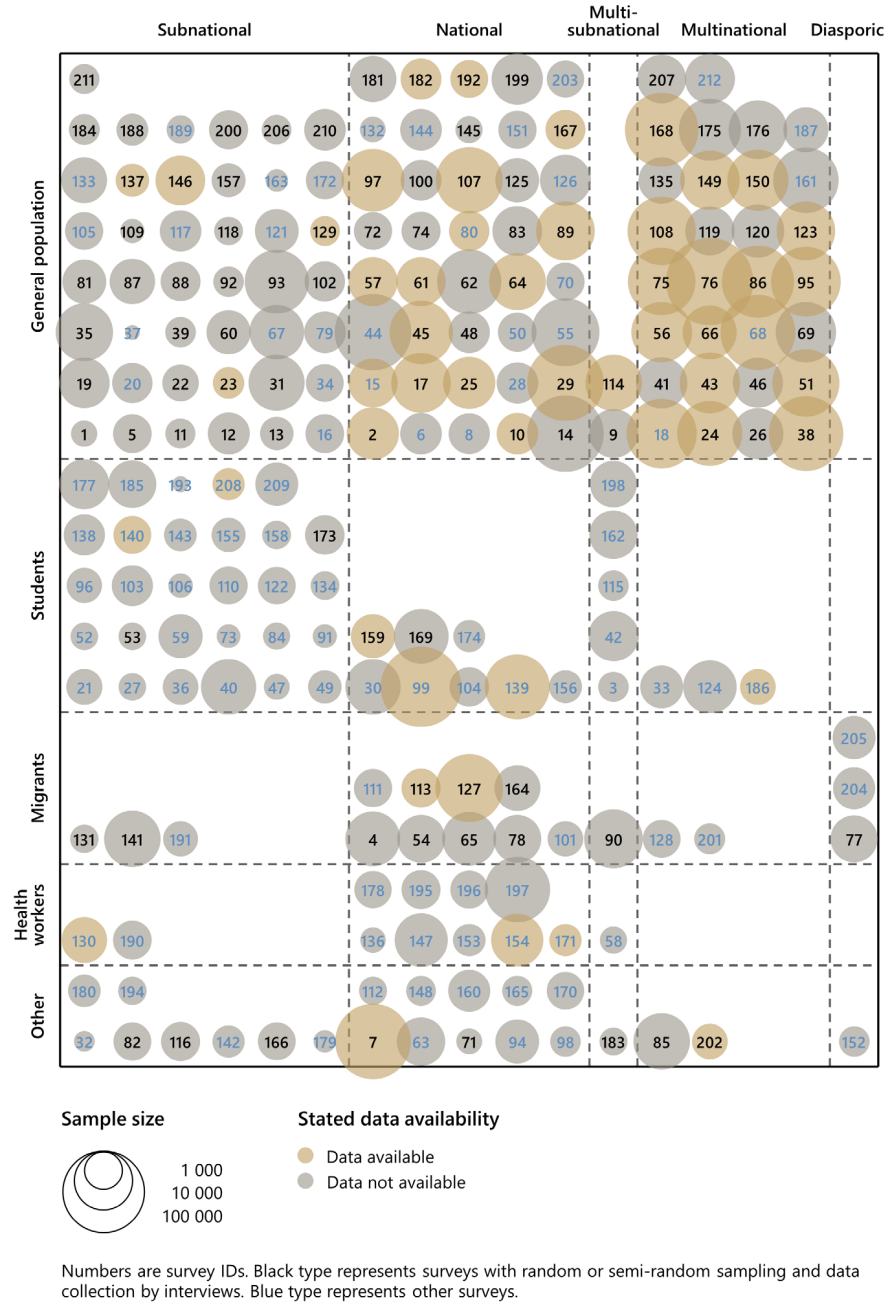
Stated data availability by survey population and geographic scale. *Note:* Numbers are survey IDs. Black type represents surveys with random or semi-random sampling and data collection by interviews. Blue type represents other surveys. In the classification of survey populations ‘students’ include health sciences students and ‘other’ include health worker migrants.

Data availability varies systematically by survey type. To illustrate, Figure 7 replicates the structure of Figure 6 and uses stated data availability instead of regional coverage. We see that data is more likely to be available for surveys of the general population as opposed to specific population groups. Moreover, stated data availability is highest for national and multinational surveys. It is only among multinational surveys of the general population that a majority of datasets are available.

## 4 Concluding remarks

We have presented a first-of-a-kind inventory of surveys that enquire about migration aspirations. For understanding migration processes, the data produced by such surveys is an essential complement to data on migration itself ( Carling & Schewel, 2018; de Haas 2021).

The work of compiling the inventory of surveys yielded two overall conclusions (1) there is a rich diversity of datasets that address migration aspirations, and (2) the standards of documentation are disappointingly low. Surprisingly often, basic information about surveys was missing from publications or difficult to obtain through the sources that were referenced. These weaknesses point to potentials for improved practice at all stages of the survey research process. In the following, we briefly summarize the implications.

When surveys are carried out, precise information about the survey design, population parameters, geographic coverage, sampling procedures, data collection method and data collection period should be documented. In the course of running the survey, such documentation might be scattered across e-mails, meetings notes, and internal memos, and require deliberate effort to compile for posterity.

When data collection has been completed, survey documentation should be made securely accessible to others. Even if the data itself remains restricted, there are rarely good reasons to limit access to metadata and documentation. An added advantage of publishing documentation and metadata is that these documents can be referred to in the methods sections of research articles, where the scope for detailed description is limited.

When research publications are written, authors should ensure that basic information about the survey – such as the parameters used in our inventory – is included. Authors should preferably also indicate where more detailed information is available. Information on data availability ought to be included regardless of whether the publication appears in a journal with a policy on data availability statements. ^6^

Survey items on migration aspirations are extremely sensitive to the exact wording, and analyses should therefore quote the question and response alternatives for key variables. This seems obvious, perhaps, but is often disregarded. Engaging actively with the wording of survey items helps ensure consistency between the data and the text. Publications should not refer to migration ‘desires’ or ‘intentions’, for instance, if the relevant survey item was phrased in terms of consideration or expectation. Similarly, publications should not infer *likelihood* to migrate from survey data on migration aspirations. Such misinterpretations are surprisingly widespread.

Regardless of the potential for better practice, the wealth of existing data represents promising opportunities now that an overview has been compiled. Information about existing data helps carry out secondary analyses, make new surveys more cost-effective, and add comparative dimensions to analyses. The inventory of surveys on migration aspirations seeks to stimulate such gains.

## Ethics and consent

Ethical approval and consent were not required

## Data Availability

All data and materials are available on Zenodo. This project contains the following underlying data: mjelva-carling-surveys-on-migration-aspirations.xlsx. (This is a dataset of metadata on surveys. It is the first comprehensive overview of existing survey data on migration aspirations, plans and intentions, with recorded metadata on geographic and temporal coverage, survey population, sample size, and other characteristics.) **DOI:** https://doi.org/10.5281/zenodo.8126542 (Mathilde Bålsrud Mjelva; Jørgen Carling March 24, 2023). Overview of surveys on migration aspirations, plans, and intentions. Data are available under the terms of the Creative Commons Attribution 4.0 International license

## Footnotes

^1^ The exact search string was TS=((aspiration* OR desire* OR intention* OR plan* OR willing* OR potential) NEAR/2 (migrat* OR emigrat*)) AND survey*.

^2^ For diasporic surveys we use the code for the country of origin (see Table 2). For surveys of migrants in a single country of destination, we use the code for the country of destination. When the geographic coverage varies across rounds in a single survey, we list all the countries that, to our knowledge, have been included in at least one round.

^3^ Six surveys are part of the Eurobarometer programme but are designed so differently that they count as separate surveys, by the criteria we applied. In contrast, the Afrobarometer and Latinobarómetro, for instance, are each counted as one multi-round survey.

^4^ A few of the multi-round surveys have an unknown total timespan. These are marked with an asterisk in the underlying data.

^5^ This was either because the documentation was insufficient or because the items on migration aspirations did not relate to the basic dimension of staying versus leaving (see Carling & Mjelva 2021).

^6^ See https://authorservices.taylorandfrancis.com/data-sharing-policies/data-availability-statements for suggested formulations that cover different situations.
