# Supplementary material for: Surveys on migration aspirations, plans and intentions: a comprehensive overview
Source: Open Res Eur. 2023 Sep 11;3:145. [Version 1] doi: 10.12688/openreseurope.15800.1 (PMC10844803; doi:10.12688/openreseurope.15800.1)
Supplement: Peer review history

# Reviewer report: Reviewer response for version 1

**DOI:** 10.21956/openreseurope.17068.r37042

**Published:** 2024-02-29

**Authors:** Elisa Barbiano di Belgiojoso

**Affiliations:** 1 Università degli Studi di Milano Bicocca, Milano, Italy

General remarks

 The study is a comprehensive review of studies dealing with migration aspirations, plans and intentions. The authors created a database of all studies on the topic by searching published articles. Each study was categorised according to the characteristics of the study itself. The paper is a useful analysis of surveys conducted since the 1960s. In my view, the paper is an important contribution to migration research on migrants' intentions, aspirations and plans.

 The paper clearly presents the design and methodological approach. The results summarise the main aspects of previous studies, taking each characteristic into account, but also combining all characteristics in the same analysis. I found the summary in Figures 6 and 7 particularly useful. The statistical analyses are descriptive (tables and maps) but accurate and the authors always provide an appropriate description of both tables and maps.

 The conclusions emphasise a crucial aspect: the need to have adequate documentation for surveys, describing all aspects (target population, design, geographical context...). The authors provide useful suggestions for other researchers.

 Some suggestions

 In my opinion, the only aspect not directly addressed in this systematic review is the distinction between first and second migration, and even more so between onward migration and return migration. This distinction would enrich the analysis.

If applicable, is the statistical analysis and its interpretation appropriate?

Yes

Is the study design appropriate and is the work technically sound?

Yes

Is the work clearly and accurately presented and does it engage with the current literature?

Yes

Are the conclusions drawn adequately supported by the results?

Yes

Are sufficient details of methods and analysis provided to allow replication by others?

Yes

Are all the source data and materials underlying the results available?

Yes

Reviewer Expertise:

Migration intentions; onward and return migration; internal mobility of migrants.

I confirm that I have read this submission and believe that I have an appropriate level of expertise to confirm that it is of an acceptable scientific standard.

# Reviewer report: Reviewer response for version 1

**DOI:** 10.21956/openreseurope.17068.r37365

**Published:** 2024-02-26

**Authors:** Luděk Jirka

**Affiliations:** 1 Univerzita Hradec Kralove, Hradec Kralove, Hradec Králové Region, Czech Republic

The article is well written; it provides a thorough and comprehensive overview of surveys on migration aspirations, and the authors deserve credit for their work. However, in my opinion, one small critical point could be emphasized. The article is based on surveys from articles published in English and in major migration journals. These journals are located in the "West" and this may limit the comprehensive focus on migration aspirations. For this reason, the article might be seen as Western-centric, as it neglects authors who had not published either in English or in major migration journals, but who still focus on migration aspirations. This is just a small caveat and I could imagine the amount of work that would be required to meet this criteria. However, the Western-centric focus also means that, for example, African scholars are under-represented (Figure 1). Authors should think about that. Furthermore, I wonder how authors could be so precise in assigning surveys to categories (Table 9) and specifications of the mindset (Table 10), when they conclude by stating that the consistency of the terminology could be questioned (sixth paragraph in conclusion). I understood that categories and specifications are more defined by the authors of this article rather than using the definitions of authors? Or authors categorise definitions of authors? I think this not entirely clear from the article(and it is important) and I recommend to authors to clearly state how they are working on this.

If applicable, is the statistical analysis and its interpretation appropriate?

Yes

Is the study design appropriate and is the work technically sound?

Yes

Is the work clearly and accurately presented and does it engage with the current literature?

Partly

Are the conclusions drawn adequately supported by the results?

Yes

Are sufficient details of methods and analysis provided to allow replication by others?

Yes

Are all the source data and materials underlying the results available?

Yes

Reviewer Expertise:

Anthropology; migration studies

I confirm that I have read this submission and believe that I have an appropriate level of expertise to confirm that it is of an acceptable scientific standard.

# Reviewer report: Reviewer response for version 1

**DOI:** 10.21956/openreseurope.17068.r37367

**Published:** 2024-02-23

**Authors:** Sandra Morgenstern

**Affiliations:** 1 University of Mannheim, Mannheim, Germany

This is a really important topic and a valuable undertaking by the authors. Although academics and practitioners rely heavily on survey data when it comes to migration, a systematic review of existing surveys on migration aspirations and related concepts is rare.  Another important contribution I see in this article is the rare in-depth focus on these surveys – particularly with regard to the survey methodology (3.4.) e.g. the sampling method or the data collection method, and the data availability (3.7.).

 Section 2: The inventory of surveys / Methodological procedure

 Given that the core of the article is to judge surveys on their methodological procedures, I would propose to be a 'role model' in the methodological section. By this I mean that I would make the methodological section more detailed and precise. I am aware that this may not be of interest to all readers but pushing for improvements in the methodology, reporting and transparency of survey research on migration goes hand in hand with being a role model.

 So I would suggest the following details: (a) a flowchart of surveys, where inclusion/exclusion goes through the search strategy, (b) more detail on inclusion criteria, e.g. in terms of language, and (c) reflections on the implications this might have (especially when thinking about paper 3: discussing biases), (d) more detail (e.g. in the flowchart) on the translation from collection of papers to collection of surveys (it is already in the text but might be confusing for readers), (e) justification for the search tool (web of science = focus/target group of academics?), ...

 Section 3: Overview of surveys

 I enjoyed reading this section. The only point I could make is that there is a lot of information: geographical coverage, temporal coverage, survey population, ... It might be easier for the reader to digest this amount of information if there was an additional (short) paragraph at the beginning of this section summarising the different focuses that follow, and one sentence why each of these is relevant / chosen as a focus. This might also be an option for the abstract, where it currently says “recorded metadata on …, and other characteristics.”, which could be too much ambiguity for some readers to read further.

 Section 4: Concluding remarks

 In this section, the authors state the following overall conclusions: “The  work  of  compiling  the  inventory  of  surveys  yielded  two overall  conclusions  (1)  there  is  a  rich  diversity  of  datasets that  address  migration  aspirations,  and  (2)  the  standards  of  documentation  are  disappointingly  low.”. I am aware that this is not a positive finding, but it is a really important one to encourage and motivate further research in this direction, particularly with regard to documentation standards and the highly interrelated quality of data. Therefore, I would suggest that these key findings be mentioned at the beginning of the article (e.g. in the abstract).

 After the conclusion, the authors move on to suggestions and recommendations for future research. Given the nature of this article in reporting facts (facts = coverage, sampling, etc.), an interpretation of the state of the art in surveys of migration aspirations would be nice at this point. For example, what are the implications of the lack of data availability? Or the non-transparent reporting of survey coverage? I see that this information is implicit in the recommendations for better research, but I think the in-between step here would be a valuable addition for readability.

 Another suggestion (and similar to the previous one, again with the intention of readability/accessibility rather than a 'must do' argument) is to link the concluding remarks back to the three key contributions made at the beginning: “First, it facilitates reuse of survey data and secondary analysis, albeit with limitations in data access, which we document. Second, it helps consolidate a sprawling field and thereby contribute to methodological and theoretical strengthening. Third, it informs debates on the ethics, politics and biases of data collection by documenting broad patterns in the body of knowledge.”. E.g. how exactly does the overview link back to ethics and/or biases?

 One minor point: An additional argument for the relevance of survey data on migration aspirations (second paragraph of the introduction) that the authors could add is that surveys collect information not only on attitudes and intended behaviour, but also on the individual's perspective on the setting and their situation.

If applicable, is the statistical analysis and its interpretation appropriate?

Not applicable

Is the study design appropriate and is the work technically sound?

Yes

Is the work clearly and accurately presented and does it engage with the current literature?

Yes

Are the conclusions drawn adequately supported by the results?

Partly

Are sufficient details of methods and analysis provided to allow replication by others?

Partly

Are all the source data and materials underlying the results available?

Yes

Reviewer Expertise:

quantitative research methods, migration research

I confirm that I have read this submission and believe that I have an appropriate level of expertise to confirm that it is of an acceptable scientific standard.

# Reviewer report: Reviewer response for version 1

**DOI:** 10.21956/openreseurope.17068.r37360

**Published:** 2024-02-05

**Authors:** Anita Brzozowska

**Affiliations:** 1 University of Warsaw, Warsaw, Poland

The article presents a noteworthy effort in providing an inventory of surveys addressing migration aspirations and related mental constructs concerning both internal and international migration. The strengths of the study lie in its attempt to create a valuable resource for researchers by compiling information on 212 surveys, offering the tool for potential extensive secondary analyses and methodological advancements. Thus, it succeeds in its primary goal of facilitating data reuse (even if the availability rate is only 25%, it can still increase the effectiveness in terms of cost and comparative dimension).

 The article provides a clear overview of the methodology employed, allowing replication. The rationale behind using the Web of Science to search survey-based literature is clearly presented, and the authors acknowledged the limitations of using such a method. I just wonder whether checking the EMM (Ethnic and Migrant Minorities) Survey Registry, a database of quantitative surveys that have been undertaken with EMM (sub)samples across Europe and beyond, would give a more detailed picture of quantitative studies conducted in underrepresented regions. Looking at Table 1, I guess no datasets addressing migration aspirations were published in data journals. Nevertheless, this option could be included in the section devoted to recommendations as an incentive to enhance documentation standards, fostering better practices in the field.

 The article could benefit from a more detailed exploration of other specific strategies addressing documentation shortcomings identified as crucial obstacles in reusing the data. It would help scholars who are not familiar with the FAIR data principles and tools like the DCC Checklist for a Data Management Plan or a 5-star deployment scheme for Open Data to navigate and discover different and often challenging aspects of open science (of course, taking into account that it is not the main topic of the manuscript).

 On another note, I am aware that survey items and questionnaire design were examined in a separate paper. However, exposing patterns and biases in the focus of the migration aspiration research would help align the article with all the ambitious objectives, namely methodological and theoretical strengthening, and informing debates on the ethics and biases of data collection. A more thorough exploration of the challenges posed by survey items and potential pitfalls in data analysis would strengthen the paper. And one minor remark of an editorial nature: there is an unnecessary note to Table 9.

 In summary, the inventory presented in the article can catalyse positive changes in research practices and facilitate data reuse.

If applicable, is the statistical analysis and its interpretation appropriate?

Not applicable

Is the study design appropriate and is the work technically sound?

Yes

Is the work clearly and accurately presented and does it engage with the current literature?

Yes

Are the conclusions drawn adequately supported by the results?

Partly

Are sufficient details of methods and analysis provided to allow replication by others?

Yes

Are all the source data and materials underlying the results available?

Yes

Reviewer Expertise:

migration in CEE, labour migration, family migration, migration aspirations

I confirm that I have read this submission and believe that I have an appropriate level of expertise to confirm that it is of an acceptable scientific standard.

# Reviewer report: Reviewer response for version 1

**DOI:** 10.21956/openreseurope.17068.r36171

**Published:** 2024-01-02

**Authors:** Ilse Ruyssen

**Affiliations:** 1 Associate Research Professor in Migration Economics at the Department of Economics, Ghent University, Ghent, Belgium

SUMMARY

 This study introduces a comprehensive inventory of surveys examining migration aspirations, plans, and intentions dating back to the 1960s. It provides essential details such as geographic focus, data collection periods, survey population, and methodologies employed. Highlighting the significance of understanding migration aspirations (used as an umbrella term) through survey data, it emphasizes their role in complementing the comprehension of migration processes and urges exploration beyond actual migration motivations. Furthermore, it underscores the relevance of migration aspirations in policy-making, particularly in addressing migration-related challenges and establishing social policy priorities. The inventory's primary goal is to facilitate researchers in locating and reusing existing survey data while exposing patterns and biases that could inform improved data collection strategies.

 The study reveals the diverse nature of quantitative datasets focusing on migration aspirations. Surveys typically involve single-round data collection at the sub-national level, predominantly in Europe or Central Asia. They primarily target the general adult population using random or semi-random sampling and predominantly employ interview-based data collection methods. The article advocates for enhanced survey documentation, secure accessibility post-data collection, and detailed descriptions in research publications. It also stresses the significance of accurately citing survey questions and response options, avoiding misinterpretation of survey data. Moreover, it encourages the utilization of existing survey data for secondary analyses, cost-effective survey designs, and comprehensive comparative studies.

 GENERAL ASSESSMENT

 This article is an extremely welcome exercise aimed ultimately at maximizing reuse of existing data by first giving it the visibility it needs (and currently lacks). More specifically, it extensively explores existing quantitative surveys on migration aspirations, delving into their significance, diversity, and implications. The resulting systematic inventory serves as an invaluable resource for researchers in the field, offering a comprehensive understanding of survey practices. Notably, the article raises crucial points regarding documentation standards in survey research, pinpointing areas for enhancement and suggesting practical solutions. Its conclusions provide clear and actionable recommendations for researchers, emphasizing detailed survey documentation, accurate data interpretation, and effective referencing, thus guiding future studies in this domain. The emphasis on leveraging existing data and datasets on migration aspirations for secondary analyses and comparative studies showcases the authors' forward-thinking approach, promoting resourceful use of available information.

 FURTHER SUGGESTIONS

 The authors mostly focus on the geographic scope, the target population and methodologies used, while giving only a few hints with respect to the survey items operationalizing the umbrella term of ‘migration aspirations’. A separate publication is planned to focus specifically on the various dimensions of such aspirations that different surveys measure via various items. However, by separating the two exercises, the ambition of the review is somewhat limited, as geographic or temporal patterns cannot be connected to dimensions of migration aspirations that are measured, which is a missed opportunity. More generally, even within the scope of this review, it may have been interesting to uncover some relationships: e.g. surveys in sub-Saharan Africa target mainly which types of population with which methods and with which methods?  

 In addition, two aspects warrant attention for their unexpected nature. Firstly, the inclusion of 'residential mobility' within the category of migration aspirations is somewhat surprising, as it diverges from the conventional focus in migration literature.

 Secondly, the incorporation of surveys among migrant populations introduces a particular dimension. Unlike surveys targeting non-migrants, these inquiries delve into aspirations to return or move onwards implicitly, and hence go beyond the typical focus on initial migration aspirations. This distinction adds complexity to the analysis of targeted populations which could be further explored. Furthermore, it could be interesting to examine whether different dimensions of migration aspirations (preference vs. consideration vs. intention) are then used in surveys that measure aspirations for onward/return migration.

 A more explicit exploration of the rationale behind these two choices would contribute to a deeper understanding of the study's methodology and enrich the overall narrative.

If applicable, is the statistical analysis and its interpretation appropriate?

Not applicable

Is the study design appropriate and is the work technically sound?

Yes

Is the work clearly and accurately presented and does it engage with the current literature?

Yes

Are the conclusions drawn adequately supported by the results?

Yes

Are sufficient details of methods and analysis provided to allow replication by others?

Yes

Are all the source data and materials underlying the results available?

Yes

Reviewer Expertise:

Migration aspirations, Staying preferences, Immobility, Determinants of migration, Implications of (imm)mobility

I confirm that I have read this submission and believe that I have an appropriate level of expertise to confirm that it is of an acceptable scientific standard.
